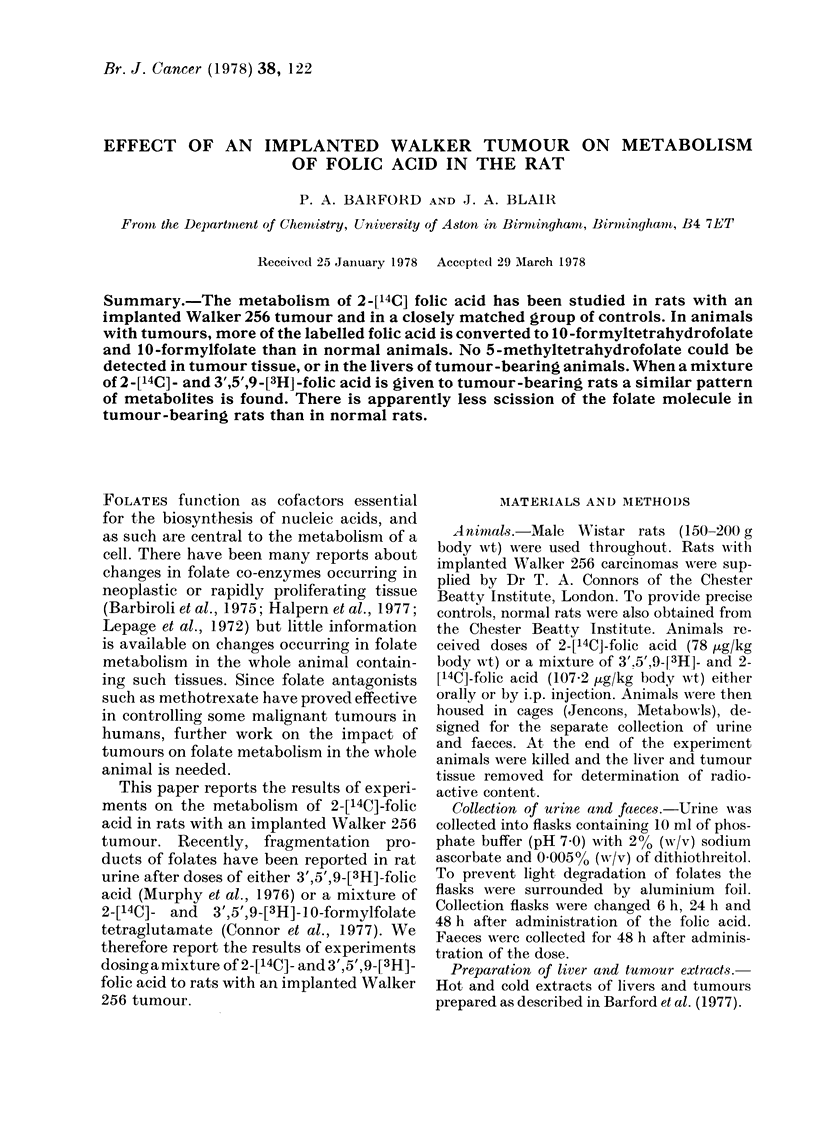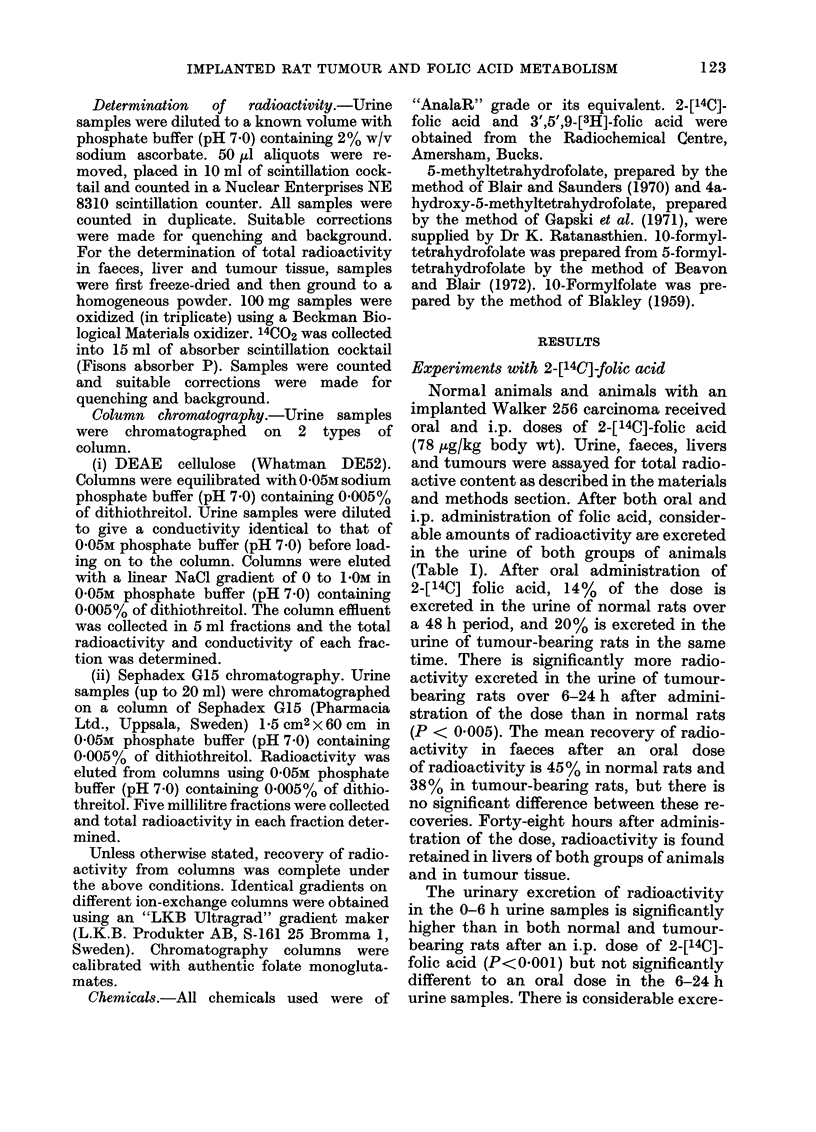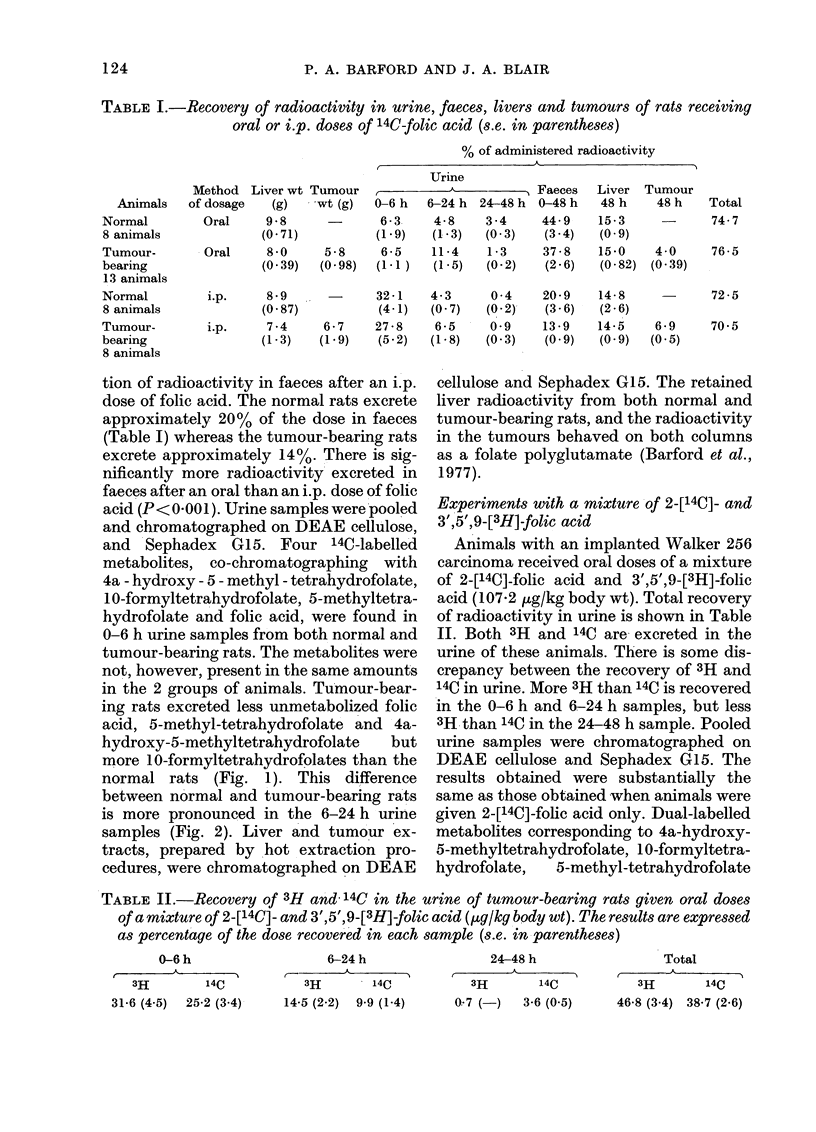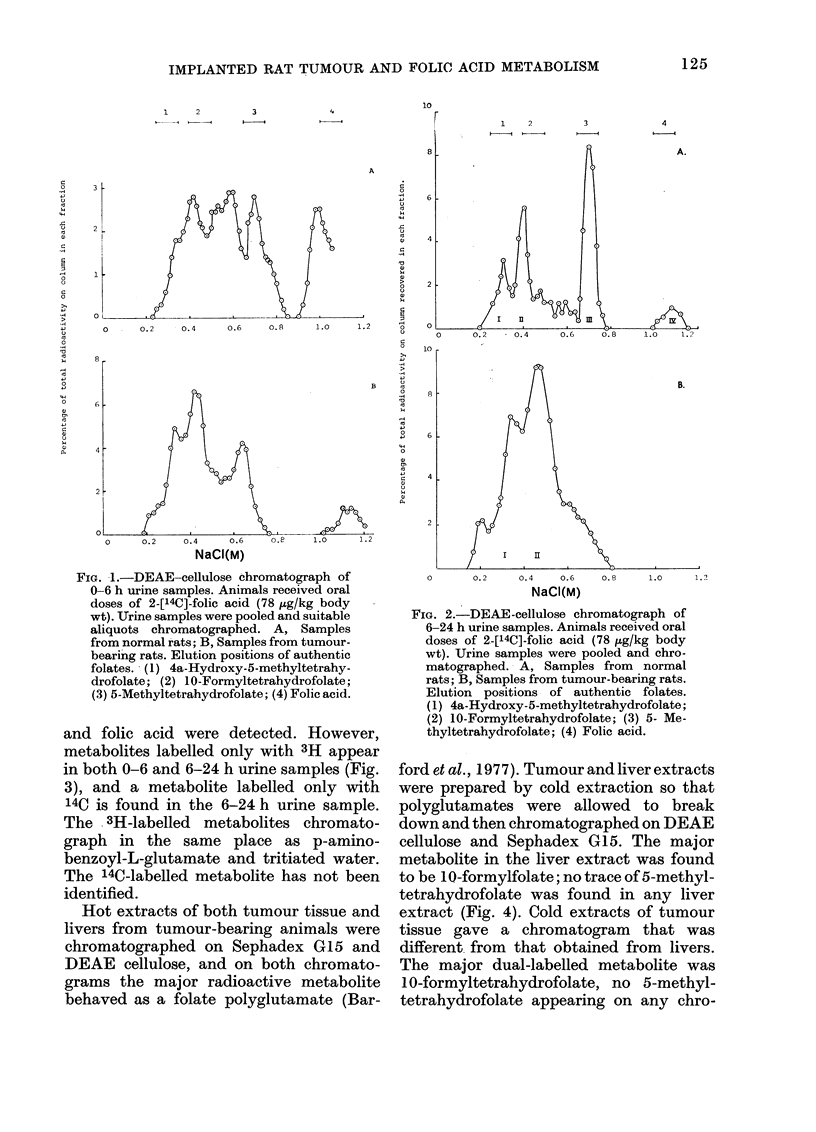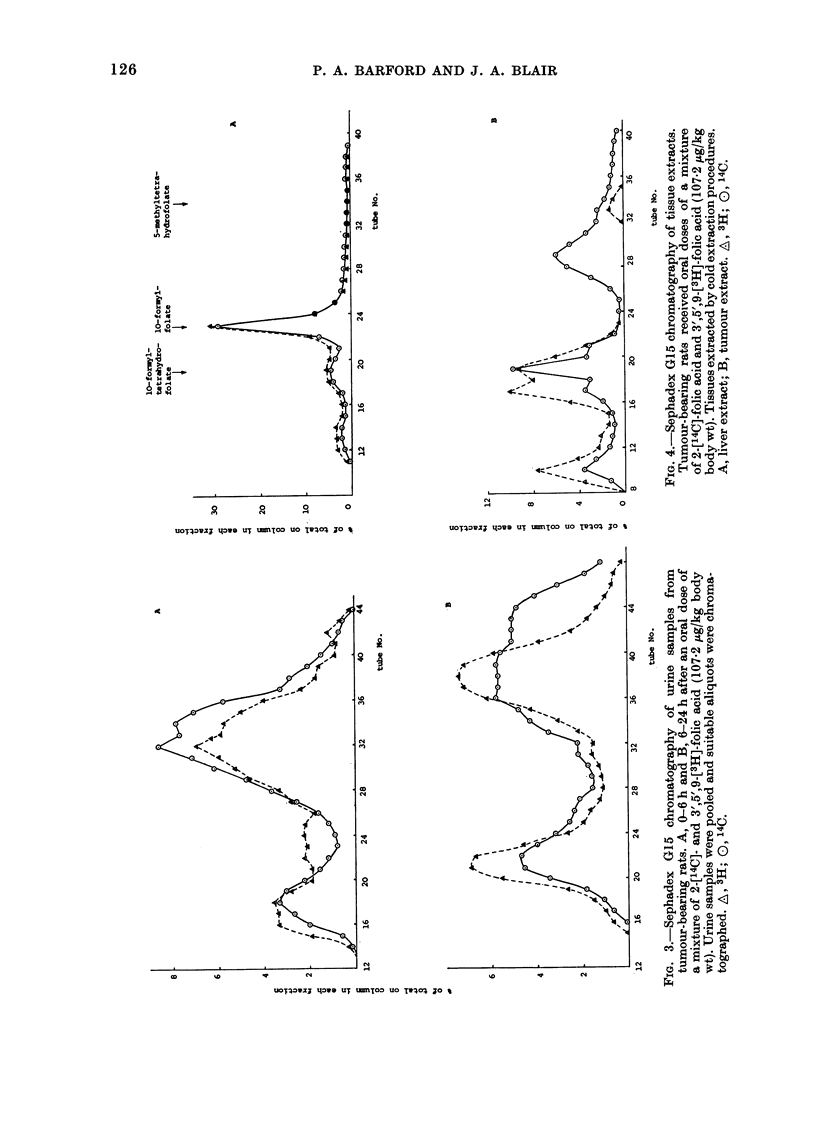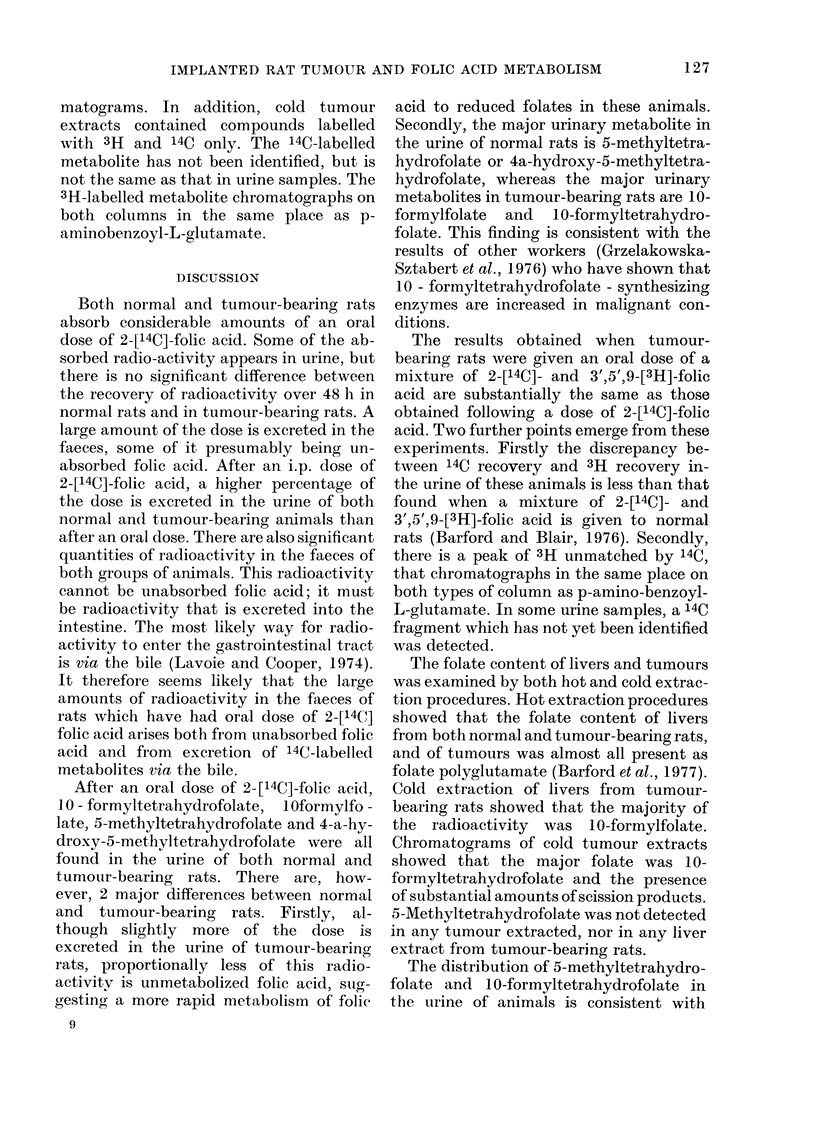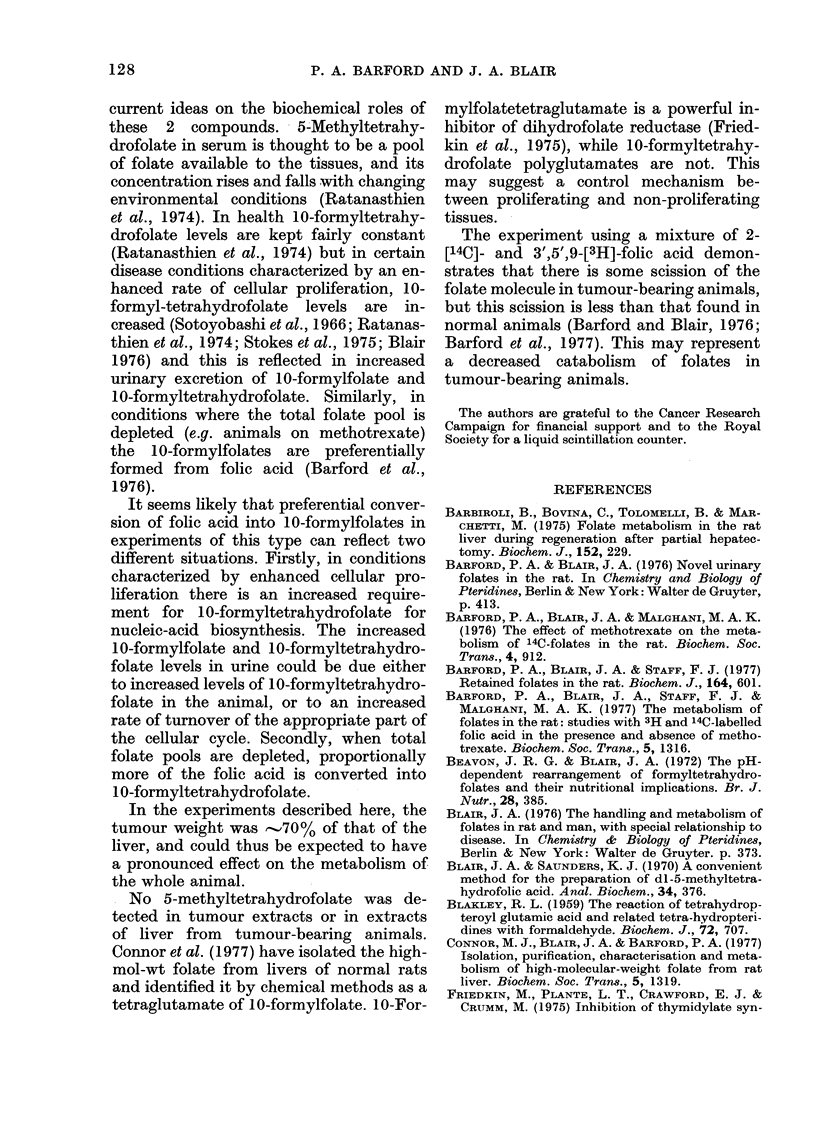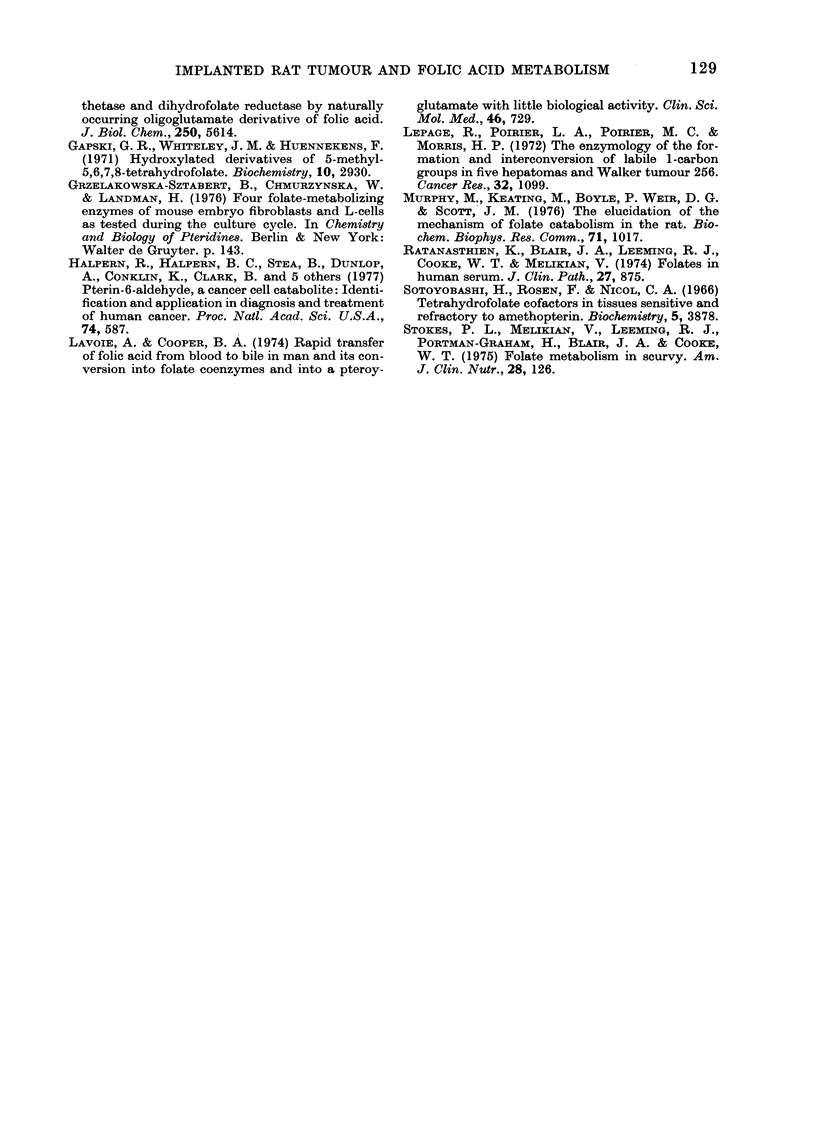# Effect of an implanted Walker tumour on metabolism of folic acid in the rat.

**DOI:** 10.1038/bjc.1978.171

**Published:** 1978-07

**Authors:** P. A. Barford, J. A. Blair

## Abstract

The metabolism of 2-[14C] folic acid has been studied in rats with an implanted Walker 256 tumour and in a closely matched group of controls. In animals with tumours, more of the labelled folic acid is converted to 10-formyltetrahydrofolate and 10-formylfolate than in normal animals. No 5-methyltetrahydrofolate could be detected in tumour tissue, or in the livers of tumour-bearing animals. When a mixture of 2-[14C]- and 3',5',9-[3H]-folic acid is given to tumour-bearing rats a similar pattern of metabolites is found. There is apparenly less scission of the folate molecule in tumour-bearing rats than in normal rats.


*Br. J. Cancer* (1978) **38**, 122

### EFFECT OF AN IMPLANTED WALKER TUMOUR ON METABOLISM OF FOLIC ACID IN THE RAT

P. A. BARFORD AND J. A. BLAIR

*From the Department of Chemistry, University of Aston in Birmingham, Birmingham, B4 7ET*

Received 25 January 1978 Accepted 29 March 1978

**Summary.—The metabolism of 2-[$^{14}$C] folic acid has been studied in rats with an implanted Walker 256 tumour and in a closely matched group of controls. In animals with tumours, more of the labelled folic acid is converted to 10-formyltetrahydrofolate and 10-formylfolate than in normal animals. No 5-methyltetrahydrofolate could be detected in tumour tissue, or in the livers of tumour-bearing animals. When a mixture of 2-[$^{14}$C]- and 3′,5′,9-[$^{3}$H]-folic acid is given to tumour-bearing rats a similar pattern of metabolites is found. There is apparently less scission of the folate molecule in tumour-bearing rats than in normal rats.**

FOLATES function as cofactors essential for the biosynthesis of nucleic acids, and as such are central to the metabolism of a cell. There have been many reports about changes in folate co-enzymes occurring in neoplastic or rapidly proliferating tissue (Barbiroli *et al.*, 1975; Halpern *et al.*, 1977; Lepage *et al.*, 1972) but little information is available on changes occurring in folate metabolism in the whole animal containing such tissues. Since folate antagonists such as methotrexate have proved effective in controlling some malignant tumours in humans, further work on the impact of tumours on folate metabolism in the whole animal is needed.

This paper reports the results of experiments on the metabolism of 2-[$^{14}$C]-folic acid in rats with an implanted Walker 256 tumour. Recently, fragmentation products of folates have been reported in rat urine after doses of either 3′,5′,9-[$^{3}$H]-folic acid (Murphy *et al.*, 1976) or a mixture of 2-[$^{14}$C]- and 3′,5′,9-[$^{3}$H]-10-formylfolate tetraglutamate (Connor *et al.*, 1977). We therefore report the results of experiments dosing a mixture of 2-[$^{14}$C]- and 3′,5′,9-[$^{3}$H]-folic acid to rats with an implanted Walker 256 tumour.

### MATERIALS AND METHODS

*Animals.*—Male Wistar rats (150–200 g body wt) were used throughout. Rats with implanted Walker 256 carcinomas were supplied by Dr T. A. Connors of the Chester Beatty Institute, London. To provide precise controls, normal rats were also obtained from the Chester Beatty Institute. Animals received doses of 2-[$^{14}$C]-folic acid (78 $\mu$g/kg body wt) or a mixture of 3′,5′,9-[$^{3}$H]- and 2-[$^{14}$C]-folic acid (107·2 $\mu$g/kg body wt) either orally or by i.p. injection. Animals were then housed in cages (Jencons, Metabowls), designed for the separate collection of urine and faeces. At the end of the experiment animals were killed and the liver and tumour tissue removed for determination of radioactive content.

*Collection of urine and faeces.*—Urine was collected into flasks containing 10 ml of phosphate buffer (pH 7·0) with 2% (w/v) sodium ascorbate and 0·005% (w/v) of dithiothreitol. To prevent light degradation of folates the flasks were surrounded by aluminium foil. Collection flasks were changed 6 h, 24 h and 48 h after administration of the folic acid. Faeces were collected for 48 h after administration of the dose.

*Preparation of liver and tumour extracts.*—Hot and cold extracts of livers and tumours prepared as described in Barford *et al.* (1977).

*Determination of radioactivity.*—Urine samples were diluted to a known volume with phosphate buffer (pH 7·0) containing 2% w/v sodium ascorbate. 50 $\mu$l aliquots were removed, placed in 10 ml of scintillation cocktail and counted in a Nuclear Enterprises NE 8310 scintillation counter. All samples were counted in duplicate. Suitable corrections were made for quenching and background. For the determination of total radioactivity in faeces, liver and tumour tissue, samples were first freeze-dried and then ground to a homogeneous powder. 100 mg samples were oxidized (in triplicate) using a Beckman Biological Materials oxidizer. $^{14}CO_2$ was collected into 15 ml of absorber scintillation cocktail (Fisons absorber P). Samples were counted and suitable corrections were made for quenching and background.

*Column chromatography.*—Urine samples were chromatographed on 2 types of column.

(i) DEAE cellulose (Whatman DE52). Columns were equilibrated with 0·05M sodium phosphate buffer (pH 7·0) containing 0·005% of dithiothreitol. Urine samples were diluted to give a conductivity identical to that of 0·05M phosphate buffer (pH 7·0) before loading on to the column. Columns were eluted with a linear NaCl gradient of 0 to 1·0M in 0·05M phosphate buffer (pH 7·0) containing 0·005% of dithiothreitol. The column effluent was collected in 5 ml fractions and the total radioactivity and conductivity of each fraction was determined.

(ii) Sephadex G15 chromatography. Urine samples (up to 20 ml) were chromatographed on a column of Sephadex G15 (Pharmacia Ltd., Uppsala, Sweden) 1·5 cm$^2$ × 60 cm in 0·05M phosphate buffer (pH 7·0) containing 0·005% of dithiothreitol. Radioactivity was eluted from columns using 0·05M phosphate buffer (pH 7·0) containing 0·005% of dithiothreitol. Five millilitre fractions were collected and total radioactivity in each fraction determined.

Unless otherwise stated, recovery of radioactivity from columns was complete under the above conditions. Identical gradients on different ion-exchange columns were obtained using an "LKB Ultragrad" gradient maker (L.K.B. Produkter AB, S-161 25 Bromma 1, Sweden). Chromatography columns were calibrated with authentic folate monoglutamates.

*Chemicals.*—All chemicals used were of "AnalaR" grade or its equivalent. 2-[$^{14}$C]-folic acid and 3′,5′,9-[$^3$H]-folic acid were obtained from the Radiochemical Centre, Amersham, Bucks.

5-methyltetrahydrofolate, prepared by the method of Blair and Saunders (1970) and 4a-hydroxy-5-methyltetrahydrofolate, prepared by the method of Gapski *et al.* (1971), were supplied by Dr K. Ratanasthien. 10-formyltetrahydrofolate was prepared from 5-formyltetrahydrofolate by the method of Beavon and Blair (1972). 10-Formylfolate was prepared by the method of Blakley (1959).

### RESULTS

### *Experiments with 2-[$^{14}$C]-folic acid*

Normal animals and animals with an implanted Walker 256 carcinoma received oral and i.p. doses of 2-[$^{14}$C]-folic acid (78 $\mu$g/kg body wt). Urine, faeces, livers and tumours were assayed for total radioactive content as described in the materials and methods section. After both oral and i.p. administration of folic acid, considerable amounts of radioactivity are excreted in the urine of both groups of animals (Table I). After oral administration of 2-[$^{14}$C] folic acid, 14% of the dose is excreted in the urine of normal rats over a 48 h period, and 20% is excreted in the urine of tumour-bearing rats in the same time. There is significantly more radioactivity excreted in the urine of tumour-bearing rats over 6–24 h after administration of the dose than in normal rats ($P < 0{\cdot}005$). The mean recovery of radioactivity in faeces after an oral dose of radioactivity is 45% in normal rats and 38% in tumour-bearing rats, but there is no significant difference between these recoveries. Forty-eight hours after administration of the dose, radioactivity is found retained in livers of both groups of animals and in tumour tissue.

The urinary excretion of radioactivity in the 0–6 h urine samples is significantly higher than in both normal and tumour-bearing rats after an i.p. dose of 2-[$^{14}$C]-folic acid ($P<0{\cdot}001$) but not significantly different to an oral dose in the 6–24 h urine samples. There is considerable excre-

TABLE I.—*Recovery of radioactivity in urine, faeces, livers and tumours of rats receiving oral or i.p. doses of* $^{14}C$*-folic acid (s.e. in parentheses)*

| | | | | % of administered radioactivity | | | | | | |
|---|---|---|---|---|---|---|---|---|---|---|
| | | | | Urine | | | | | | |
| Animals | Method of dosage | Liver wt (g) | Tumour wt (g) | 0–6 h | 6–24 h | 24–48 h | Faeces 0–48 h | Liver 48 h | Tumour 48 h | Total |
| Normal 8 animals | Oral | 9·8 (0·71) | — | 6·3 (1·9) | 4·8 (1·3) | 3·4 (0·3) | 44·9 (3·4) | 15·3 (0·9) | — | 74·7 |
| Tumour-bearing 13 animals | Oral | 8·0 (0·39) | 5·8 (0·98) | 6·5 (1·1) | 11·4 (1·5) | 1·3 (0·2) | 37·8 (2·6) | 15·0 (0·82) | 4·0 (0·39) | 76·5 |
| Normal 8 animals | i.p. | 8·9 (0·87) | — | 32·1 (4·1) | 4·3 (0·7) | 0·4 (0·2) | 20·9 (3·6) | 14·8 (2·6) | — | 72·5 |
| Tumour-bearing 8 animals | i.p. | 7·4 (1·3) | 6·7 (1·9) | 27·8 (5·2) | 6·5 (1·8) | 0·9 (0·3) | 13·9 (0·9) | 14·5 (0·9) | 6·9 (0·5) | 70·5 |

tion of radioactivity in faeces after an i.p. dose of folic acid. The normal rats excrete approximately 20% of the dose in faeces (Table I) whereas the tumour-bearing rats excrete approximately 14%. There is significantly more radioactivity excreted in faeces after an oral than an i.p. dose of folic acid ($P<0{\cdot}001$). Urine samples were pooled and chromatographed on DEAE cellulose, and Sephadex G15. Four $^{14}C$-labelled metabolites, co-chromatographing with 4a-hydroxy-5-methyl-tetrahydrofolate, 10-formyltetrahydrofolate, 5-methyltetrahydrofolate and folic acid, were found in 0–6 h urine samples from both normal and tumour-bearing rats. The metabolites were not, however, present in the same amounts in the 2 groups of animals. Tumour-bearing rats excreted less unmetabolized folic acid, 5-methyl-tetrahydrofolate and 4a-hydroxy-5-methyltetrahydrofolate but more 10-formyltetrahydrofolates than the normal rats (Fig. 1). This difference between normal and tumour-bearing rats is more pronounced in the 6–24 h urine samples (Fig. 2). Liver and tumour extracts, prepared by hot extraction procedures, were chromatographed on DEAE cellulose and Sephadex G15. The retained liver radioactivity from both normal and tumour-bearing rats, and the radioactivity in the tumours behaved on both columns as a folate polyglutamate (Barford *et al.*, 1977).

*Experiments with a mixture of* 2-[$^{14}$C]- *and* 3′,5′,9-[$^{3}H$]*-folic acid*

Animals with an implanted Walker 256 carcinoma received oral doses of a mixture of 2-[$^{14}$C]-folic acid and 3′,5′,9-[$^{3}$H]-folic acid (107·2 μg/kg body wt). Total recovery of radioactivity in urine is shown in Table II. Both $^{3}$H and $^{14}$C are excreted in the urine of these animals. There is some discrepancy between the recovery of $^{3}$H and $^{14}$C in urine. More $^{3}$H than $^{14}$C is recovered in the 0–6 h and 6–24 h samples, but less $^{3}$H than $^{14}$C in the 24–48 h sample. Pooled urine samples were chromatographed on DEAE cellulose and Sephadex G15. The results obtained were substantially the same as those obtained when animals were given 2-[$^{14}$C]-folic acid only. Dual-labelled metabolites corresponding to 4a-hydroxy-5-methyltetrahydrofolate, 10-formyltetrahydrofolate, 5-methyl-tetrahydrofolate

TABLE II.—*Recovery of* $^{3}H$ *and* $^{14}C$ *in the urine of tumour-bearing rats given oral doses of a mixture of* 2-[$^{14}C$]- *and* 3′,5′,9-[$^{3}H$]*-folic acid (μg/kg body wt). The results are expressed as percentage of the dose recovered in each sample (s.e. in parentheses)*

| 0–6 h | | 6–24 h | | 24–48 h | | Total | |
|---|---|---|---|---|---|---|---|
| $^{3}$H | $^{14}$C | $^{3}$H | $^{14}$C | $^{3}$H | $^{14}$C | $^{3}$H | $^{14}$C |
| 31·6 (4·5) | 25·2 (3·4) | 14·5 (2·2) | 9·9 (1·4) | 0·7 (—) | 3·6 (0·5) | 46·8 (3·4) | 38·7 (2·6) |

FIG. 1.—DEAE-cellulose chromatograph of 0–6 h urine samples. Animals received oral doses of 2-[$^{14}$C]-folic acid (78 $\mu$g/kg body wt). Urine samples were pooled and suitable aliquots chromatographed. A, Samples from normal rats; B, Samples from tumour-bearing rats. Elution positions of authentic folates. (1) 4a-Hydroxy-5-methyltetrahydrofolate; (2) 10-Formyltetrahydrofolate; (3) 5-Methyltetrahydrofolate; (4) Folic acid.

FIG. 2.—DEAE-cellulose chromatograph of 6–24 h urine samples. Animals received oral doses of 2-[$^{14}$C]-folic acid (78 $\mu$g/kg body wt). Urine samples were pooled and chromatographed. A, Samples from normal rats; B, Samples from tumour-bearing rats. Elution positions of authentic folates. (1) 4a-Hydroxy-5-methyltetrahydrofolate; (2) 10-Formyltetrahydrofolate; (3) 5- Methyltetrahydrofolate; (4) Folic acid.

and folic acid were detected. However, metabolites labelled only with $^{3}$H appear in both 0–6 and 6–24 h urine samples (Fig. 3), and a metabolite labelled only with $^{14}$C is found in the 6–24 h urine sample. The $^{3}$H-labelled metabolites chromatograph in the same place as p-aminobenzoyl-L-glutamate and tritiated water. The $^{14}$C-labelled metabolite has not been identified.

Hot extracts of both tumour tissue and livers from tumour-bearing animals were chromatographed on Sephadex G15 and DEAE cellulose, and on both chromatograms the major radioactive metabolite behaved as a folate polyglutamate (Barford *et al.*, 1977). Tumour and liver extracts were prepared by cold extraction so that polyglutamates were allowed to break down and then chromatographed on DEAE cellulose and Sephadex G15. The major metabolite in the liver extract was found to be 10-formylfolate; no trace of 5-methyltetrahydrofolate was found in any liver extract (Fig. 4). Cold extracts of tumour tissue gave a chromatogram that was different from that obtained from livers. The major dual-labelled metabolite was 10-formyltetrahydrofolate, no 5-methyltetrahydrofolate appearing on any chromatograms. In addition, cold tumour extracts contained compounds labelled with $^{3}H$ and $^{14}C$ only. The $^{14}C$-labelled metabolite has not been identified, but is not the same as that in urine samples. The $^{3}H$-labelled metabolite chromatographs on both columns in the same place as p-aminobenzoyl-L-glutamate.

### DISCUSSION

Both normal and tumour-bearing rats absorb considerable amounts of an oral dose of 2-[$^{14}C$]-folic acid. Some of the absorbed radio-activity appears in urine, but there is no significant difference between the recovery of radioactivity over 48 h in normal rats and in tumour-bearing rats. A large amount of the dose is excreted in the faeces, some of it presumably being unabsorbed folic acid. After an i.p. dose of 2-[$^{14}C$]-folic acid, a higher percentage of the dose is excreted in the urine of both normal and tumour-bearing animals than after an oral dose. There are also significant quantities of radioactivity in the faeces of both groups of animals. This radioactivity cannot be unabsorbed folic acid; it must be radioactivity that is excreted into the intestine. The most likely way for radioactivity to enter the gastrointestinal tract is *via* the bile (Lavoie and Cooper, 1974). It therefore seems likely that the large amounts of radioactivity in the faeces of rats which have had oral dose of 2-[$^{14}C$] folic acid arises both from unabsorbed folic acid and from excretion of $^{14}C$-labelled metabolites *via* the bile.

After an oral dose of 2-[$^{14}C$]-folic acid, 10-formyltetrahydrofolate, 10formylfolate, 5-methyltetrahydrofolate and 4-a-hydroxy-5-methyltetrahydrofolate were all found in the urine of both normal and tumour-bearing rats. There are, however, 2 major differences between normal and tumour-bearing rats. Firstly, although slightly more of the dose is excreted in the urine of tumour-bearing rats, proportionally less of this radioactivity is unmetabolized folic acid, suggesting a more rapid metabolism of folic acid to reduced folates in these animals. Secondly, the major urinary metabolite in the urine of normal rats is 5-methyltetrahydrofolate or 4a-hydroxy-5-methyltetrahydrofolate, whereas the major urinary metabolites in tumour-bearing rats are 10-formylfolate and 10-formyltetrahydrofolate. This finding is consistent with the results of other workers (Grzelakowska-Sztabert *et al.*, 1976) who have shown that 10-formyltetrahydrofolate-synthesizing enzymes are increased in malignant conditions.

The results obtained when tumour-bearing rats were given an oral dose of a mixture of 2-[$^{14}C$]- and 3′,5′,9-[$^{3}H$]-folic acid are substantially the same as those obtained following a dose of 2-[$^{14}C$]-folic acid. Two further points emerge from these experiments. Firstly the discrepancy between $^{14}C$ recovery and $^{3}H$ recovery in the urine of these animals is less than that found when a mixture of 2-[$^{14}C$]- and 3′,5′,9-[$^{3}H$]-folic acid is given to normal rats (Barford and Blair, 1976). Secondly, there is a peak of $^{3}H$ unmatched by $^{14}C$, that chromatographs in the same place on both types of column as p-amino-benzoyl-L-glutamate. In some urine samples, a $^{14}C$ fragment which has not yet been identified was detected.

The folate content of livers and tumours was examined by both hot and cold extraction procedures. Hot extraction procedures showed that the folate content of livers from both normal and tumour-bearing rats, and of tumours was almost all present as folate polyglutamate (Barford *et al.*, 1977). Cold extraction of livers from tumour-bearing rats showed that the majority of the radioactivity was 10-formylfolate. Chromatograms of cold tumour extracts showed that the major folate was 10-formyltetrahydrofolate and the presence of substantial amounts of scission products. 5-Methyltetrahydrofolate was not detected in any tumour extracted, nor in any liver extract from tumour-bearing rats.

The distribution of 5-methyltetrahydrofolate and 10-formyltetrahydrofolate in the urine of animals is consistent with current ideas on the biochemical roles of these 2 compounds. 5-Methyltetrahydrofolate in serum is thought to be a pool of folate available to the tissues, and its concentration rises and falls with changing environmental conditions (Ratanasthien *et al.*, 1974). In health 10-formyltetrahydrofolate levels are kept fairly constant (Ratanasthien *et al.*, 1974) but in certain disease conditions characterized by an enhanced rate of cellular proliferation, 10-formyl-tetrahydrofolate levels are increased (Sotoyobashi *et al.*, 1966; Ratanasthien *et al.*, 1974; Stokes *et al.*, 1975; Blair 1976) and this is reflected in increased urinary excretion of 10-formylfolate and 10-formyltetrahydrofolate. Similarly, in conditions where the total folate pool is depleted (*e.g.* animals on methotrexate) the 10-formylfolates are preferentially formed from folic acid (Barford *et al.*, 1976).

It seems likely that preferential conversion of folic acid into 10-formylfolates in experiments of this type can reflect two different situations. Firstly, in conditions characterized by enhanced cellular proliferation there is an increased requirement for 10-formyltetrahydrofolate for nucleic-acid biosynthesis. The increased 10-formylfolate and 10-formyltetrahydrofolate levels in urine could be due either to increased levels of 10-formyltetrahydrofolate in the animal, or to an increased rate of turnover of the appropriate part of the cellular cycle. Secondly, when total folate pools are depleted, proportionally more of the folic acid is converted into 10-formyltetrahydrofolate.

In the experiments described here, the tumour weight was ~70% of that of the liver, and could thus be expected to have a pronounced effect on the metabolism of the whole animal.

No 5-methyltetrahydrofolate was detected in tumour extracts or in extracts of liver from tumour-bearing animals. Connor *et al.* (1977) have isolated the high-mol-wt folate from livers of normal rats and identified it by chemical methods as a tetraglutamate of 10-formylfolate. 10-Formylfolatetetraglutamate is a powerful inhibitor of dihydrofolate reductase (Friedkin *et al.*, 1975), while 10-formyltetrahydrofolate polyglutamates are not. This may suggest a control mechanism between proliferating and non-proliferating tissues.

The experiment using a mixture of 2-[$^{14}$C]- and 3′,5′,9-[$^{3}$H]-folic acid demonstrates that there is some scission of the folate molecule in tumour-bearing animals, but this scission is less than that found in normal animals (Barford and Blair, 1976; Barford *et al.*, 1977). This may represent a decreased catabolism of folates in tumour-bearing animals.

The authors are grateful to the Cancer Research Campaign for financial support and to the Royal Society for a liquid scintillation counter.

### REFERENCES

Barbiroli, B., Bovina, C., Tolomelli, B. & Marchetti, M. (1975) Folate metabolism in the rat liver during regeneration after partial hepatectomy. *Biochem. J.*, **152**, 229.

Barford, P. A. & Blair, J. A. (1976) Novel urinary folates in the rat. In *Chemistry and Biology of Pteridines*, Berlin & New York: Walter de Gruyter, p. 413.

Barford, P. A., Blair, J. A. & Malghani, M. A. K. (1976) The effect of methotrexate on the metabolism of $^{14}$C-folates in the rat. *Biochem. Soc. Trans.*, **4**, 912.

Barford, P. A., Blair, J. A. & Staff, F. J. (1977) Retained folates in the rat. *Biochem. J.*, **164**, 601.

Barford, P. A., Blair, J. A., Staff, F. J. & Malghani, M. A. K. (1977) The metabolism of folates in the rat: studies with $^{3}$H and $^{14}$C-labelled folic acid in the presence and absence of methotrexate. *Biochem. Soc. Trans.*, **5**, 1316.

Beavon, J. R. G. & Blair, J. A. (1972) The pH-dependent rearrangement of formyltetrahydrofolates and their nutritional implications. *Br. J. Nutr.*, **28**, 385.

Blair, J. A. (1976) The handling and metabolism of folates in rat and man, with special relationship to disease. In *Chemistry & Biology of Pteridines*, Berlin & New York: Walter de Gruyter. p. 373.

Blair, J. A. & Saunders, K. J. (1970) A convenient method for the preparation of dl-5-methyltetrahydrofolic acid. *Anal. Biochem.*, **34**, 376.

Blakley, R. L. (1959) The reaction of tetrahydropteroyl glutamic acid and related tetra-hydropteridines with formaldehyde. *Biochem. J.*, **72**, 707.

Connor, M. J., Blair, J. A. & Barford, P. A. (1977) Isolation, purification, characterisation and metabolism of high-molecular-weight folate from rat liver. *Biochem. Soc. Trans.*, **5**, 1319.

Friedkin, M., Plante, L. T., Crawford, E. J. & Crumm, M. (1975) Inhibition of thymidylate synthetase and dihydrofolate reductase by naturally occurring oligoglutamate derivative of folic acid. *J. Biol. Chem.*, **250**, 5614.

GAPSKI, G. R., WHITELEY, J. M. & HUENNEKENS, F. (1971) Hydroxylated derivatives of 5-methyl-5,6,7,8-tetrahydrofolate. *Biochemistry*, **10**, 2930.

GRZELAKOWSKA-SZTABERT, B., CHMURZYNSKA, W. & LANDMAN, H. (1976) Four folate-metabolizing enzymes of mouse embryo fibroblasts and L-cells as tested during the culture cycle. In *Chemistry and Biology of Pteridines*. Berlin & New York: Walter de Gruyter. p. 143.

HALPERN, R., HALPERN, B. C., STEA, B., DUNLOP, A., CONKLIN, K., CLARK, B. and 5 others (1977) Pterin-6-aldehyde, a cancer cell catabolite: Identification and application in diagnosis and treatment of human cancer. *Proc. Natl. Acad. Sci. U.S.A.*, **74**, 587.

LAVOIE, A. & COOPER, B. A. (1974) Rapid transfer of folic acid from blood to bile in man and its conversion into folate coenzymes and into a pteroyglutamate with little biological activity. *Clin. Sci. Mol. Med.*, **46**, 729.

LEPAGE, R., POIRIER, L. A., POIRIER, M. C. & MORRIS, H. P. (1972) The enzymology of the formation and interconversion of labile 1-carbon groups in five hepatomas and Walker tumour 256. *Cancer Res.*, **32**, 1099.

MURPHY, M., KEATING, M., BOYLE, P. WEIR, D. G. & SCOTT, J. M. (1976) The elucidation of the mechanism of folate catabolism in the rat. *Biochem. Biophys. Res. Comm.*, **71**, 1017.

RATANASTHIEN, K., BLAIR, J. A., LEEMING, R. J., COOKE, W. T. & MELIKIAN, V. (1974) Folates in human serum. *J. Clin. Path.*, **27**, 875.

SOTOYOBASHI, H., ROSEN, F. & NICOL, C. A. (1966) Tetrahydrofolate cofactors in tissues sensitive and refractory to amethopterin. *Biochemistry*, **5**, 3878.

STOKES, P. L., MELIKIAN, V., LEEMING, R. J., PORTMAN-GRAHAM, H., BLAIR, J. A. & COOKE, W. T. (1975) Folate metabolism in scurvy. *Am. J. Clin. Nutr.*, **28**, 126.